# PYGL-mediated glucose metabolism reprogramming promotes EMT phenotype and metastasis of pancreatic cancer

**DOI:** 10.7150/ijbs.76756

**Published:** 2023-03-21

**Authors:** Qian Ji, Hengchao Li, Zhiwei Cai, Xiao Yuan, Xi Pu, Yumeng Huang, Shengqiao Fu, Liangmei Chu, Chongyi Jiang, Junli Xue, Xiaoxin Zhang, Rongkun Li

**Affiliations:** 1 Department of Radiation Oncology, Institute of Oncology, Affiliated Hospital of Jiangsu University, Zhenjiang 212001, China.; 2 Department of Pancreatic surgery, Huashan Hospital, Fudan University, Shanghai 200040, China.; 3 Department of General Surgery, Hepato-biliary-pancreatic Center, Huadong Hospital, Fudan University, Shanghai 200040, China.; 4 Department of Burn and Plastic Surgery, Affiliated Hospital of Jiangsu University, Zhenjiang 212001, China.; 5 Department of Oncology, Shanghai East Hospital, School of Medicine, Tongji University, Shanghai 200123, China.; 6 Jiangsu Key Laboratory of Medical Science and Laboratory Medicine, School of Medicine, Jiangsu University, Zhenjiang 212013, China.

**Keywords:** PDAC, Hypoxia, PYGL, Glucose metabolism reprogramming, EMT, Metastasis

## Abstract

Epithelial-mesenchymal transition (EMT) is closely associated with tumor invasion and metastasis. However, key regulators of EMT in pancreatic ductal adenocarcinoma (PDAC) need to be further studied. Bioinformatics analyses of pancreatic cancer public datasets showed that glycogen phosphorylase L (PYGL) expression is elevated in quasimesenchymal PDAC (QM-PDAC) and positively associated with EMT. *In vitro* cellular experiments further confirm PYGL as a crucial EMT regulator in PDAC cells. Functionally, PYGL overexpression promotes cell migration and invasion *in vitro* and facilitates liver metastasis *in vivo*, while PYGL knockdown has opposite effects. Mechanically, hypoxia induces PYGL expression in a hypoxia inducible factor 1α (HIF1α)-dependent manner and promotes glycogen accumulation. Elevated PYGL mobilizes accumulated glycogen to fuel glycolysis via its activity as a glycogen phosphorylase, thus inducing the EMT process, which could be suppressed by the glycolysis inhibitor 2-deoxy-D-glucose (2-DG). Clinically, PYGL expression is upregulated in PDAC and correlates with its malignant features and poor prognosis. Collectively, the data from our study reveal that the hypoxia/PYGL/glycolysis-induced EMT promotes PDAC metastasis, which establishes the rational for targeting hypoxia/PYGL/glycolysis/EMT signaling pathway against PDAC.

## Introduction

Cancer metastasis is a complicated multistep cellular biological process 1,2, which takes responsibility for more than 90% of cancer-related death from various human solid malignancies, including pancreatic ductal adenocarcinoma (PDAC). Despite encouraging progress in the diagnosis and therapy of PDAC, the prognosis for PDAC patients is still poor, with a 5-year survival rate around 8% 3. So far, the only potentially curative treatment for PDAC is surgical resection, while more than 80% of PDAC patients are not eligible for surgery due to diagnosis in advanced stages with local invasion or distant metastasis caused by the lack of clinically informative early diagnostic symptom and biomarkers 4. Hence, exploring the primary molecules and mechanisms underlying PDAC progression is urgently needed.

Epithelial-mesenchymal transition (EMT) is well-known to play a crucial role in tumor malignant progression by facilitating tumor cell invasion and dissemination to distant organs, thereby resulting in cancer metastasis 5. Although the role of EMT in cancer has attracted intensive attention, key regulators of EMT remain largely unknown. Therefore, identifying crucial regulators of EMT could provide opportunities to develop novel therapeutic strategies for suppressing metastasis and thus improving treatment outcome. Here, we analyzed the differentially expressed genes between classical PDAC and quasimesenchymal PDAC (QM-PDAC) tissues from the Cancer Genome Atlas (TCGA) and Gene Expression Omnibus (GEO) datasets (GSE15471, GSE16515, and GSE17891), and found that the expression of glycogen phosphorylase L (PYGL) was significantly increased in QM-PDAC tissues compared with that in classical PDAC. The results from Gene Set Enrichment Analysis (GSEA) further confirmed a positive correlation between *PYGL* expression and EMT. Thus, our study is to decipher the molecular events regulating EMT and metastasis mediated by PYGL in PDAC.

Glycogen comprises a store of glucose and is mainly present in liver and muscles. The glycogen structure consists of long polymetric chains of glucose units linked by α-1,4 glycosidic bonds with occasional α-1,6 glycosidic bonds that generate branching points and increase solubility 6. Glycogen synthesis and breakdown involves the activity of several enzymes and regulatory proteins, among which glycogen synthases (GSs) and glycogen phosphorylases (GPs) catalyze the key steps of synthesis and breakdown, respectively 6. GSs elongate glycogen branches through the formation of α-1,4 glycosidic bridges, which are cleaved by GPs to release glucose-1-phosphate (G1P) that enters the glycolytic pathway or the pentose phosphate (PPP) pathway by conversion to glucose-6-phosphate (G6P) 7. Glycogen synthase has two isoforms: GYS1 (muscle) and GYS2 (liver), while glycogen phosphorylase has three isoforms: PYGM (muscle), PYGL (liver), and PYGB (brain). Increasing evidence indicates that glycogen metabolism plays key roles in various cancers via many glycogen-related enzymes. Recent studies have largely focused on PYGB regarding the roles of GPs in cancer. However, few studies on the roles of PYGL in cancer are so far available, and PYGL has an unclear function in PDAC.

Cancer cells undergo metabolic reprogramming in response to hypoxic microenvironment present in solid tumors. It has been well documented that glycogen accumulation and glycogen-related enzyme expression increase under hypoxic conditions in both noncancer and cancer cell lines 8-11. More importantly, cancer cells exhibit a high rate of glycolysis even in the presence of oxygen, which is known as the Warburg effect or aerobic glycolysis. Cancer cells rely primarily on aerobic glycolysis, which could produce ATP as well as glycolytic intermediates for biosynthesis of nucleotides, lipids, and proteins to support cancer growth 12. Simultaneously, the majority of glucose is converted to lactate, which facilitates tumor invasion and metastasis via acidifying the tumor microenvironment 13, 14. Numerous studies have shown the enhancement of glycolysis induced by oncogenic factors via various mechanisms in solid tumors.

In the current study, we discovered that hypoxia induces PYGL expression in a hypoxia inducible factor 1α (HIF1α)-dependent manner and increases glycogen accumulation. Elevated PYGL fuels accumulated glycogen into glycolysis as a glycogen phosphorylase, thereby inducing EMT and facilitating tumor metastasis. PYGL expression is increased in PDAC tissues and positively associated with prognosis for PDAC patients. Taken together, the data from our study identify PYGL as a regulator of EMT to promote PDAC metastasis by mobilizing glycogen to fuel glycolysis and may provide a potential new therapeutic target for this devastating disease.

## Results

### Integrated analysis identifies EMT-associated proteins in PDAC

**Figure 1:**
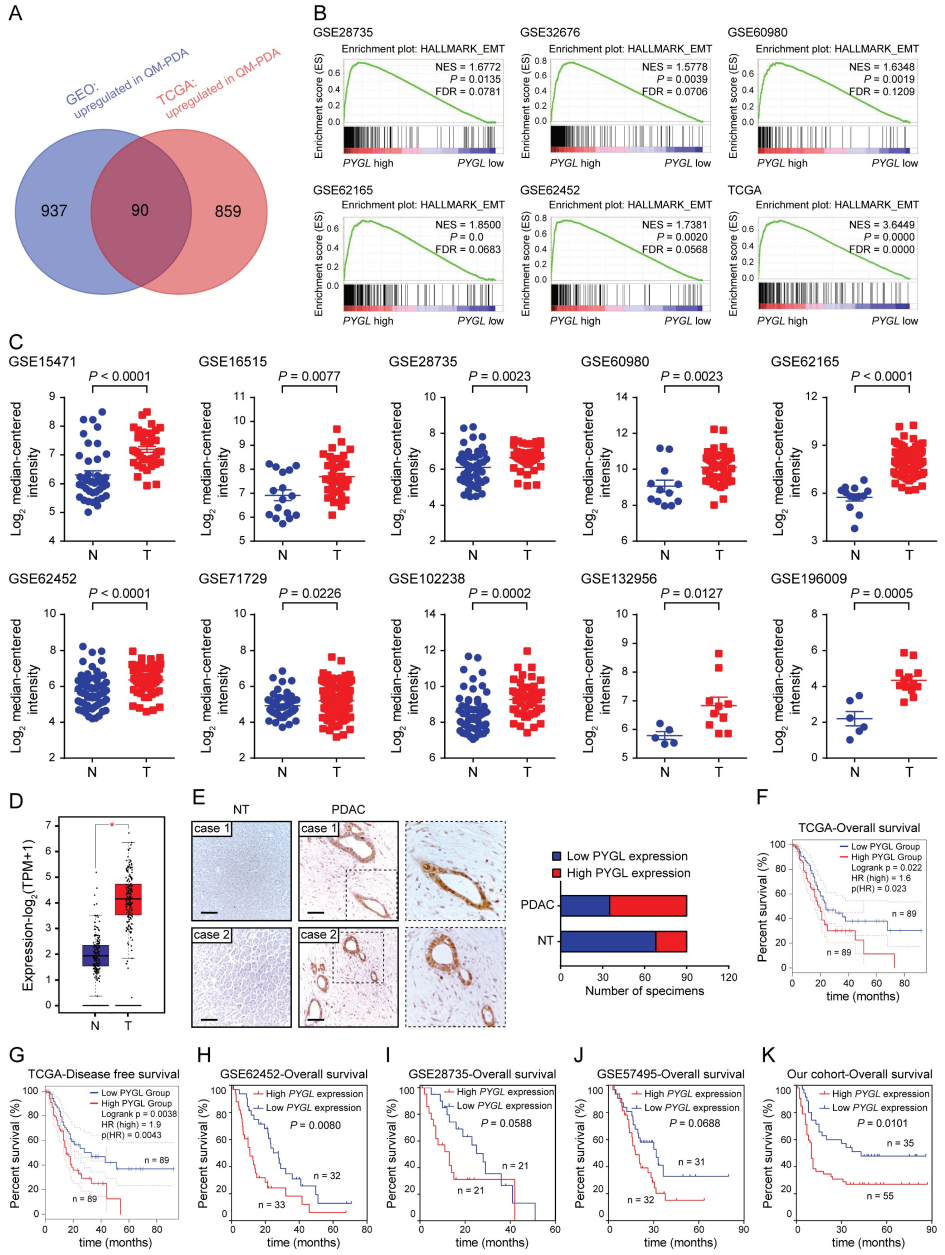
Identifying critical proteins in pancreatic cancer EMT. (A) Venn diagram showed 90 upregulated genes in mesenchymal subtypes compared with epithelial subtypes in the GEO (GSE15471、GSE16515, and GSE17819) and TCGA datasets. (B) Positive correlation between high *PYGL* mRNA expression and glycolysis revealed by Gene Set Enrichment Analysis (GSEA) using hallmark gene sets. (C) *PYGL* mRNA expression in PDAC tissues (T) and normal pancreas tissues (N) by analyzing multiple gene expression profiles from the GEO database. (D) *PYGL* mRNA expression in PDAC tissues (T) and normal pancreas tissues (N) by analyzing the data derived from the TCGA cohort and GTEx. (E) PYGL protein expression in the matched non-tumor (NT) tissues and PDAC tissues in tissue microarray revealed by IHC staining and representative IHC images of PYGL in NT and PDAC tissues. Scale bar, 50 μm. (F-G) Comparison of overall survival (F) and disease-free survival (G) of PDAC patients with different *PYGL* mRNA expression in the TCGA cohort. (H-J) Comparison of overall survival of PDAC patients with different *PYGL* mRNA expression in the GSE62452 (H), GSE28735 (I), and GSE57495 (J). (K) Comparison of overall survival of PDAC patients with different PYGL protein expression in our cohort.

To determine the factors contributing to EMT in PDAC, data from the GEO and TCGA datasets were obtained and stratified into epithelial and mesenchymal subtypes as previously defined (Figure S1A-B) 15. In the spectrum of 90 protein coding genes that were significantly overexpressed in the mesenchymal subtypes compared with the epithelial subtypes in both the GEO and TCGA datasets (Figure 1A), several differentially expressed genes (DEGs) were known EMT-related factors (*TGFB1*, *SNAI2*, *FN1*, *CDH2*, *LOX*, and *TWIST1*), indicating that our analysis was built on meaningful data in the context of EMT. We then investigated the clinical relevance of these DEGs using the TCGA database and found that only 16 of them positively correlated with a poor prognosis of PDAC (4 with* P* value < 0.01 and 12 with *P* value < 0.05) (Table S1). *PYGL* was selected for further investigation due to its unknown roles in EMT and significant prognostic value in PDAC. GSEA of the GEO and TCGA datasets was further performed based on the *PYGL* mRNA expression level and revealed that *PYGL* expression was significantly associated with EMT signature (Figure 1B). We also analyzed the correlation between* PYGL* expression and EMT markers at mRNA level using the TCGA database. Accordingly, *PYGL* was strongly associated with almost all EMT markers, including *CDH2*, *VIM*, *MMP2*, *MMP9*, *FN*, *SNAI1*, *SNAI2*, *ZEB1*, *ZEB2*, *TWIST1*, and *TWIST2* (Figure S2). The data above suggest that PYGL may be involved in the process of EMT in PDAC.

### PYGL expression is increased in PDAC tissues and correlates with poor prognosis

Next, we tested PYGL expression and its clinical relevance in human PDAC tissues. Interrogating multiple GEO datasets showed higher *PYGL* mRNA expression in PDAC tissues compared with normal pancreas tissues (Figure 1C). Similar result was obtained in the TCGA-PAAD dataset (Figure 1D). Then, immunohistochemistry (IHC) staining of tissue microarray was performed and confirmed that high PYGL protein expression was detected in 55/90 of pancreatic tissues, but only in 22/90 of the adjacent non-tumor tissues (Figure 1E). IHC staining also showed that PYGL was predominantly localized in the cytoplasm and nucleus of both cancer cells (Figure 1E) and normal pancreas cells (Figure S3).

To determine the clinical significance of PYGL in PDAC, PDAC samples with follow-up information from TCGA and GEO databases were utilized to evaluate the prognostic significance of PYGL expression. The results from the TCGA database showed that PDAC patients with high *PYGL* expression had much worse overall survival (Figure 1F) and disease-free survival (Figure 1G) than those with low *PYGL* expression. The results from the GSE62452 dataset also showed that PDAC patients with high *PYGL* expression had much worse overall survival than those with low *PYGL* expression (Figure 1H). The same trends were observed in the GSE28735 (Figure 1I) and GSE57495 (Figure 1J) datasets, even if there were no significant differences. In our cohort, we found that high PYGL expression was significantly associated with lager tumor size, frequent vascular invasion, and advanced TNM stages as shown in Table S2. The survival analysis of the follow-up information showed much better overall survival in PDAC patients with low PYGL expression, although the proportion of such patients was relatively low (Figure 1K). Overall, the above results indicate that high PYGL expression is correlated with more aggressive behaviors in PDAC patients and predicts a poor prognosis.

### PYGL is a crucial EMT regulator of PDAC cells

**Figure 2:**
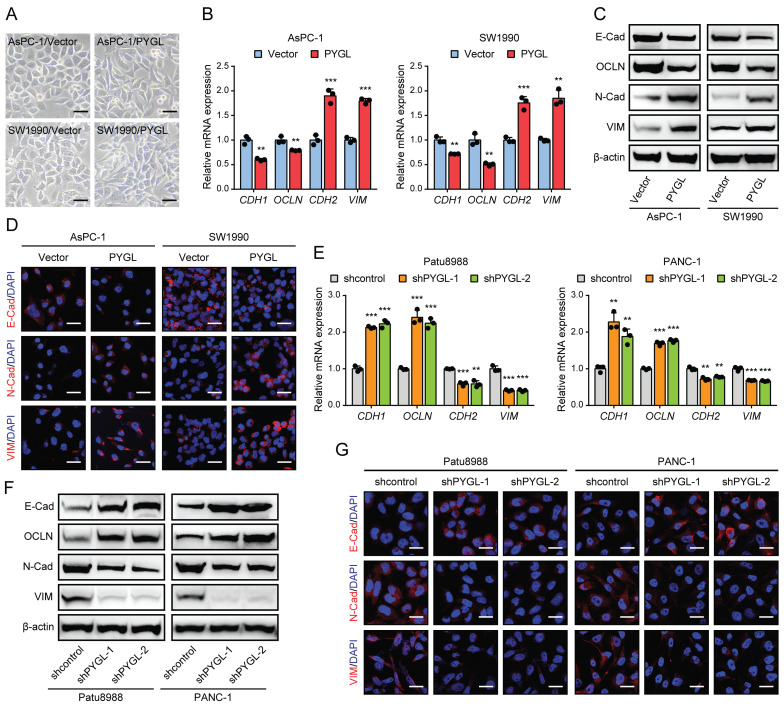
PYGL induces EMT in PDAC cells. (A) Morphology changes of AsPC-1 and SW1990 following by PYGL overexpression (scale bar, 50 μm). (B) The mRNA expression levels of E-cadherin (*CDH1*), Occludin (*OCLN*), N-cadherin (*CDH2*), and Vimentin (*VIM*) following by PYGL overexpression as revealed by qRT-PCR. (C) The protein expression levels of E-cadherin (E-Cad), Occludin (OCLN), N-cadherin (N-Cad), and Vimentin (VIM) following by PYGL overexpression as revealed by western blotting. (D) Immunofluorescence (IF) analyses of E-Cad, N-Cad, and VIM (in red) following by PYGL overexpression (scale bar, 25 μm). (E-G) The expression of EMT markers following by PYGL knockdown in Patu8988 and PANC-1 cells as revealed by qRT-PCR (E), western blotting (F), and IF staining (G) (G: scale bar, 25 μm). **P < 0.01 and ***P < 0.001.

To determine whether PYGL regulates the EMT process of PDAC, we first examined PYGL expression in human PDAC cells and an immortalized normal pancreatic ductal cell (HPNE) by quantitative real-time PCR (qRT-PCR) (Figure S4A) and western blotting (Figure S4B). It was noticed that PYGL expression of HPNE cell was lower than that of the majority of PDAC cells at protein level (Figure S4B). AsPC-1 and SW1990 cells with lower PYGL expression were selected for overexpression, while Patu8988 and PANC-1 cells with higher PYGL expression for knockdown. The efficiency of overexpression or knockdown was confirmed by qRT-PCR and western blotting (Figure S4C-F). PYGL shRNAs with higher knockdown efficiency, shPYGL-1 and shPYGL-2, were used in further study. We also examined the expression of other components of the glycogen metabolism, including critical synthetic enzymes (GYS1 and GBE1) and catabolic enzymes (PYGB and PYGM), and found that neither PYGL overexpression nor knockdown had obvious effects on their expression as evidenced by western blotting (Figure S4G-H). Indeed, we observed a mesenchymal-like phenotype in AsPC-1 and SW1990 cells followed by PYGL overexpression (Figure 2A). qRT-PCR and western blotting assays showed that PYGL overexpression led to a decreased expression of EMT epithelial markers (E-Cadherin and Occludin), but an increased expression of EMT mesenchymal markers (N-Cadherin and Vimentin) (Figure 2B-C). Furthermore, Immunofluorescence (IF) analysis validated the effects of PYGL overexpression on EMT (Figure 2D). On the contrary, PYGL knockdown exhibited the opposite effects on EMT, with decreased expression of E-Cadherin and Occludin and increased expression of N-Cadherin and Vimentin revealed by qRT-PCR (Figure 2E), western blotting (Figure 2F), and IF analysis (Figure 2G). Collectively, the results indicate that PYGL is a positive regulator for PDAC EMT.

### PYGL promotes PDAC cell invasion *in vitro* and tumor metastasis *in vivo*

**Figure 3:**
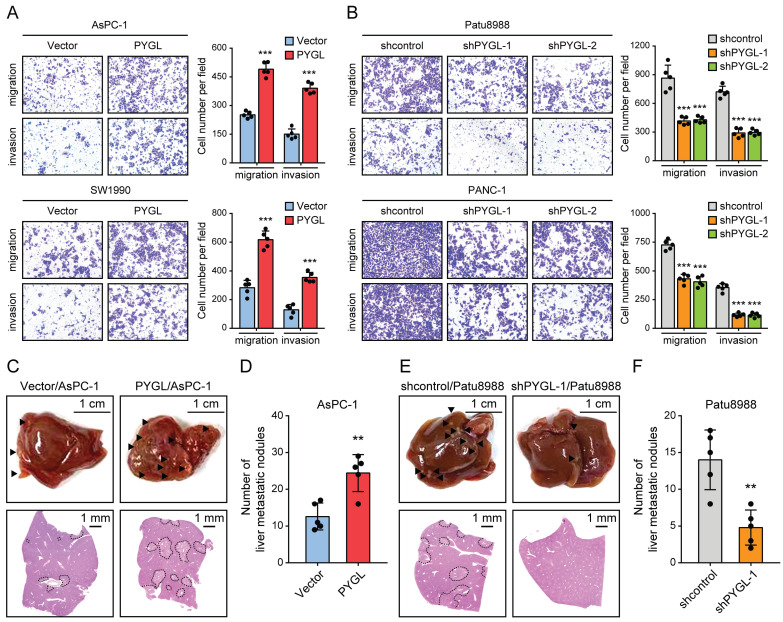
PYGL facilitates cell migration and invasion *in vitro* and liver metastasis *in vivo*. (A) The promoting effects of PYGL overexpression on cell migration and invasion confirmed by Transwell assays (magnification, 100 ×). (B) The inhibitory effects of PYGL knockdown on cell migration and invasion confirmed by Transwell assays (magnification, 100 ×). (C) Representative images of liver metastatic nodules of intrasplenic xenografts from PYGL-overexpressing and vector control AsPC-1 cells (▲, liver metastasis lesion; upper panel) and corresponding hematoxylin and eosin (H&E) images (bottom panel). (D) Quantification of the liver metastatic nodules in (C). (E) Representative images of liver metastatic nodules of intrasplenic xenografts from PYGL knockdown and control Patu8988 cells (▲, liver metastasis lesion; upper panel) and corresponding H&E images (bottom panel). (F) Quantification of the liver metastatic nodules in (E). **P < 0.01 and ***P < 0.001.

To discover the roles of PYGL in PDAC progression, we examined the effects of PYGL on cell migration and invasion. As a result, PYGL overexpression significantly promoted cell migration and invasion of AsPC-1and SW1990 (Figure 3A), whereas PYGL knockdown inhibited cell migration and invasion of Patu8988 and PANC-1 (Figure 3B), as revealed by Transwell migration and invasion assays. In addition, PYGL overexpression significantly enhanced the proliferative capacity of AsPC-1 and SW1990 (Figure S5A), while PYGL knockdown had the opposite effects on cell proliferation of Patu8988 and PANC-1 (Figure S5B), as revealed by CCK-8 and colony formation assays. But inconsistent with the results of a previous study 8, the activity of senescence-associated β-galactosidase (SA-β-gal), a well-established marker for cellular senescence, was not affected by PYGL knockdown (Figure S5C).

Given that EMT is closely with tumor invasion and metastasis, we therefore focused our analyses on tumor invasion and metastasis in the following study. In order to verify the *in vitro* results from Tranwell migration and invasion assays, we investigated the effects of PYGL on tumor metastasis *in vivo* using liver metastasis model via intrasplenic injection. As expected, the results from liver metastasis model showed that mice injected with PYGL overexpression cells had more liver metastatic nodules compared with the control group (Figure 3C-D). In contrast, PYGL knockdown had the opposite effects on liver metastasis (Figure 3E-F). Taken together, these data indicate the oncogenic roles of PYGL in PDAC development and progression.

### PYGL enzymatic activity is required for the induction of EMT and malignant phenotype

**Figure 4:**
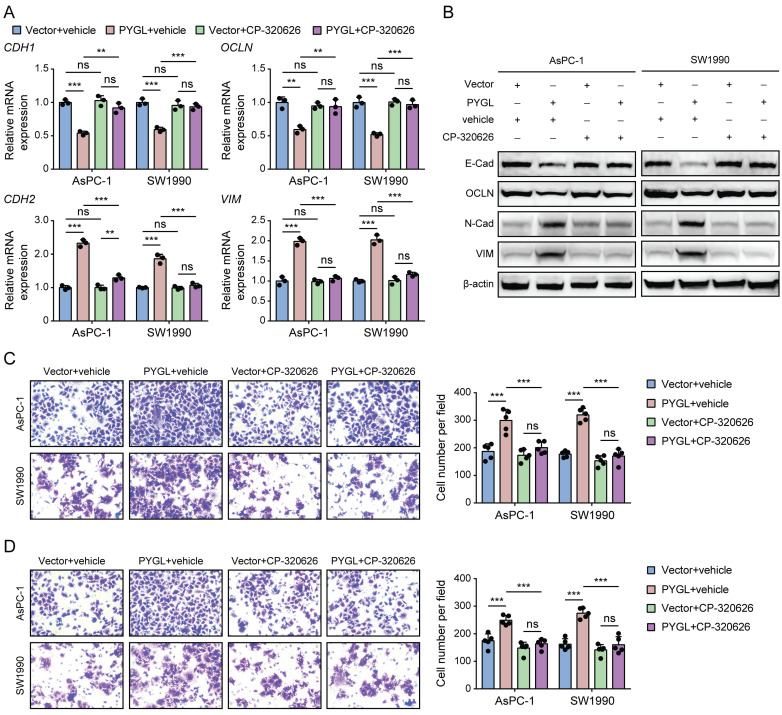
The catalytic activity is responsible for the promoting effects of PYGL on EMT, cell migration, and invasion. (A) The mRNA expression of EMT markers (*CDH1*, *OCLN*, *CDH2*, and *VIM*) in PYGL-overexpressing and vector control cells treated with or without CP-320626 (25 μM for 24 hours) examined by qRT-PCR. (B) The protein expression of EMT markers (E-Cad, OCLN, N-Cad, and VIM) in PYGL-overexpressing and vector control cells treated with or without CP-320626 (25 μM for 24 hours) examined by western blotting. (C-D) Transwell migration (C) and invasion (D) assays of PYGL-overexpressing and vector control cells treated with or without 25 μM CP-320626 (magnification, 200 ×). ns, no significance, **P < 0.01, and ***P < 0.001.

Previous studies have reported that CP-320626, a glycogen phosphorylase inhibitor, inhibits pancreatic cancer cell proliferation and induces cell cycle arrest and apoptosis 16, 17. So, we examined whether its enzymatic activity is required for PYGL to induce EMT using CP-320626 treatment. Loss of PYGL enzymatic activity by CP-320626 did recover the decreased expression of E-cadherin and Occludin and abolished the increased expression of N-cadherin and Vimentin induced by PYGL overexpression revealed by qRT-PCR (Figure 4A) and western blotting (Figure 4B), indicating that PYGL induces EMT dependent on its enzymatic activity. Furthermore, we observed that CP-320626 dramatically attenuated the promoting effects of PYGL overexpression on cell migration (Figure 4C) and invasion (Figure 4D). Therefore, PYGL enzymatic activity is essential for EMT, cell migration, and invasion.

### Glycogen mobilization stimulated by PYGL fuels glycolysis in PDAC cells

**Figure 5:**
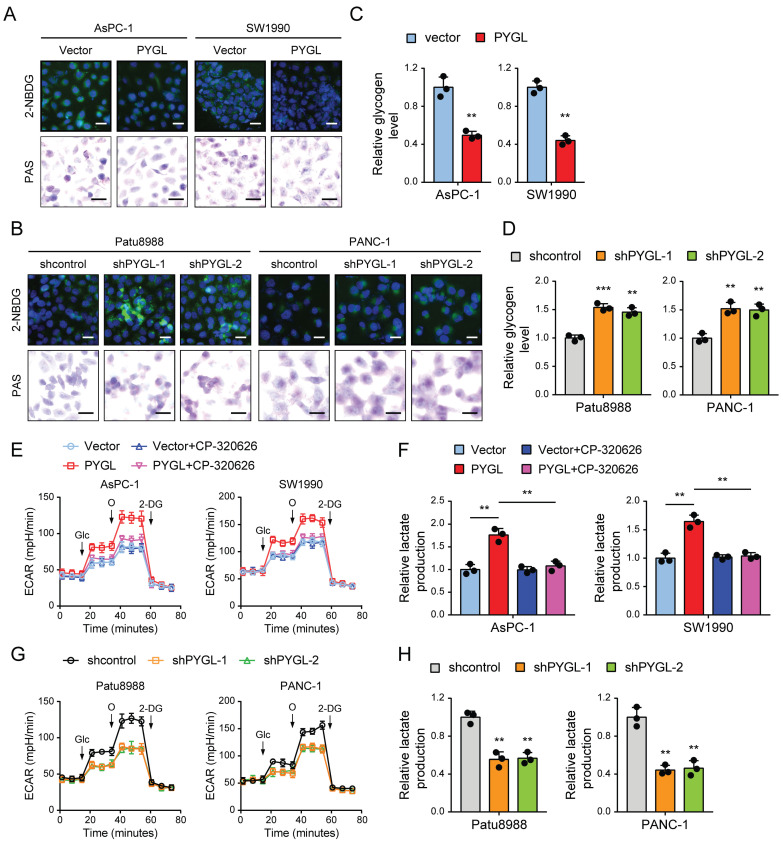
PYGL mobilized glycogen to fuel glycolysis. (A) Glycogen was visualized by 2-NBDG fluorescence and PAS staining in PYGL-overexpressing and vector control cells. (B) Glycogen was visualized by 2-NBDG fluorescence and PAS staining in PYGL knockdown and control cells. (C) Glycogen assay of PYGL-overexpressing and vector control cells. (D) Glycogen assay of PYGL knockdown and control cells. (E) Glycolysis of PYGL-overexpressing and vector control cells reflected by extracellular acidification rates (ECAR) was measured using the Seahorse SF96 Extracellular Flux Analyzer (Glc, glucose; O, oligomycin). (F) Lactate production in PYGL-overexpressing and vector control cells. (G) Glycolysis of PYGL knockdown and control cells reflected by ECAR was measured using the Seahorse SF96 Extracellular Flux Analyzer (Glc, glucose; O, oligomycin). (H) Lactate production in PYGL knockdown and control cells. (scale bar of 2-NBDG fluorescence in A and B, 100 μm; scale bar of PAS staining in A and B, 20 μm). **P < 0.01 and ***P < 0.001.

Based on the above results, we speculated that PYGL might potentially catalyze glycogen breakdown in PDAC cells. To confirm it, we evaluated the effect of PYGL on glycogen content in PDAC cells by periodic acid Schiff (PAS) staining and the conversion of a fluorescent glucose analog 2-(N-(7-nitrobenz-2-oxa-1,3-diazol-4-yl) amino)-2-deoxyglucose (2-NBDG) into glycogen granules. Expectedly, PYGL overexpression showed significantly reduced glycogen content in AsPC-1 and SW1990 cells (Figure 5A). Conversely, PYGL knockdown showed significantly increased glycogen content in Patu8988 and PANC-1 cells (Figure 5B). The results were further confirmed by measuring the glycogen level with a Glycogen Assay Kit as shown in Figure 5C-D. These data further indicate that PYGL exerts its enzymatic activity in pancreatic cancer cells.

Glycogen breakdown by GPs causes the release of G1P that is then converted to G-6-P by the enzyme phosphoglucomutase, and G6P enters the glycolytic cascade or the PPP pathway 18. PYGL has been shown to function as glycogen enzyme to regulate the glycolysis pathway, thereby exerting its oncogenic roles 19. Therefore, the effects of PYGL on glycolysis were tested in PDAC cells. We first investigated PDCA cell dependency on glycolysis by measuring the extracellular acidification rate (ECAR) with a real-time metabolite analyzer and lactate production with a BioVision kit. In line with the previous study, PYGL overexpression increased the ECAR and lactate production of AsPC-1 and SW1990 cells (Figure 5E-F), whereas PYGL knockdown had the opposite effects in Patu8988 and PANC-1 cells (Figure 5G-H). Furthermore, we observed that CP-320626 could abolish the promoting effects of PYGL on ECAR and lactate production (Figure 5E-F). Treatment with CP-320626 did not affect the basal levels of ECAR or lactate production in AsPC-1 and SW1990 cells (Figure 5E-F). Inconsistent with these observations, CP-320626 significantly attenuated the basal levels of ECAR in Patu8988 and PANC-1 cells (Figure S6), possibly due to the different PYGL expression levels among the four PDAC cells. Collectively, these data suggest that PYGL is a positive regulator for the glycolysis pathway dependent on its catalytic activity.

### Enhanced glycolysis is required for the effects of PYGL on PDAC EMT and malignant phenotypes

Next, we evaluated the functional role of glycolysis for PYGL-mediated regulation of PDAC cell EMT, migration, and invasion. To this end, we treated the cells with 2-deoxy-D-glucose (2-DG), a glycolytic inhibitor, to block the glycolytic pathway. Indeed, the altered expression of EMT markers induced by PYGL overexpression was eliminated upon treatment with 2-DG as evidenced by qRT-PCR (Figure S7A) and western blotting (Figure S7B). Moreover, the PYGL-mediated increase in cell migration and invasion was largely abrogated by 2-DG treatment (Figure S7C-D). These data strongly suggest that increased glycolysis is critical for PYGL to facilitate PDAC EMT and progression, which can be targeted by glycolysis inhibition.

### PYGL expression is induced by hypoxia in a HIF1α-dependent fashion in PDAC cells

**Figure 6:**
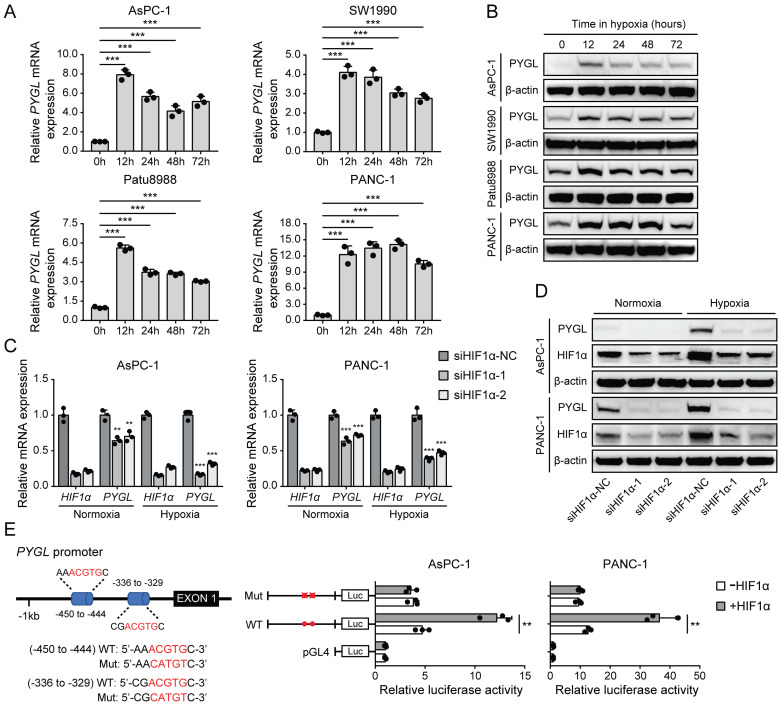
PYGL expression is induced by hypoxia as a HIF1α target. (A) *PYGL* mRNA expression in PDAC cells exposed to hypoxia for 0-72 hours examined by qRT-PCR. (B) PYGL protein expression in PDAC cells 0-72 hours examined by western blotting. (C-D) The expression of PYGL and HIF1α at mRNA (C) and protein (D) levels in AsPC-1 and PANC-1 cells transiently transfected with siRNAs targeting HIF1α exposed to normoxia or hypoxia for 12 hours. (E) Luciferase analysis in AsPC-1 and PANC-1 cells. The cells overexpressing HIF1α expression plasmid (+HIF1α) or control vector pcDNA3.1(+) (-HIF1α) were transfected with the pGL4-PYGL promoter or pGL4-PYGL promoter mutation. 48 hours after transfection, the cells were subjected to dual luciferase analysis. Red sites represent the putative HIF1α-binding sites; Mut, mutant; WT, wild-type. **P < 0.01 and ***P < 0.001.

It has been shown that several glycogen breakdown enzymes, including PYGL, are induced in hypoxic conditions 8, 9. Thus, we examined whether PYGL expression is induced in hypoxia in PDAC cells. In PDAC cell lines used for PYGL overexpression and knockdown, PYGL expression was remarkably increased at mRNA (Figure 6A) and protein (Figure 6B) levels under hypoxic conditions. Next, we used siRNAs targeting HIF1α to determine whether hypoxia induces PYGL expression in a HIF1α-dependent manner or not. The results showed that HIF1α depletion resulted in a significantly reduced PYGL expression in AsPC-1 and PANC-1 cells at mRNA (Figure 6C) and protein (Figure 6D) levels under both normoxic and hypoxic conditions, indicating that PYGL expression is induced by hypoxia in a HIF1α-dependent manner. To understand the underlying mechanism of PYGL overexpression in hypoxia, we surveyed the promotor region of the PYGL gene and identified two hypoxia response elements (HREs) (Figure 6E). Therefore, we constructed a full-length PYGL luciferase promotor vector construct (containing two HERs) to investigate whether the binding of HIF1α activates PYGL promotor. As shown in Figure 6E, HIF1α could significantly increase the activity of PYGL promotor in AsPC-1 and PANC-1 cells. To verify whether HREs is required for HIF1α to transactivate PYGL promotor, we mutated these two HREs simultaneously. As expected, the mutation almost completely abolished the transactivation of PYGL promotor mediated by HIF1α. Collectively, the data suggest that hypoxia induces PYGL expression dependent on HIF1α.

### Elevated PYGL mobilizes accumulated glycogen in hypoxic conditions

**Figure 7:**
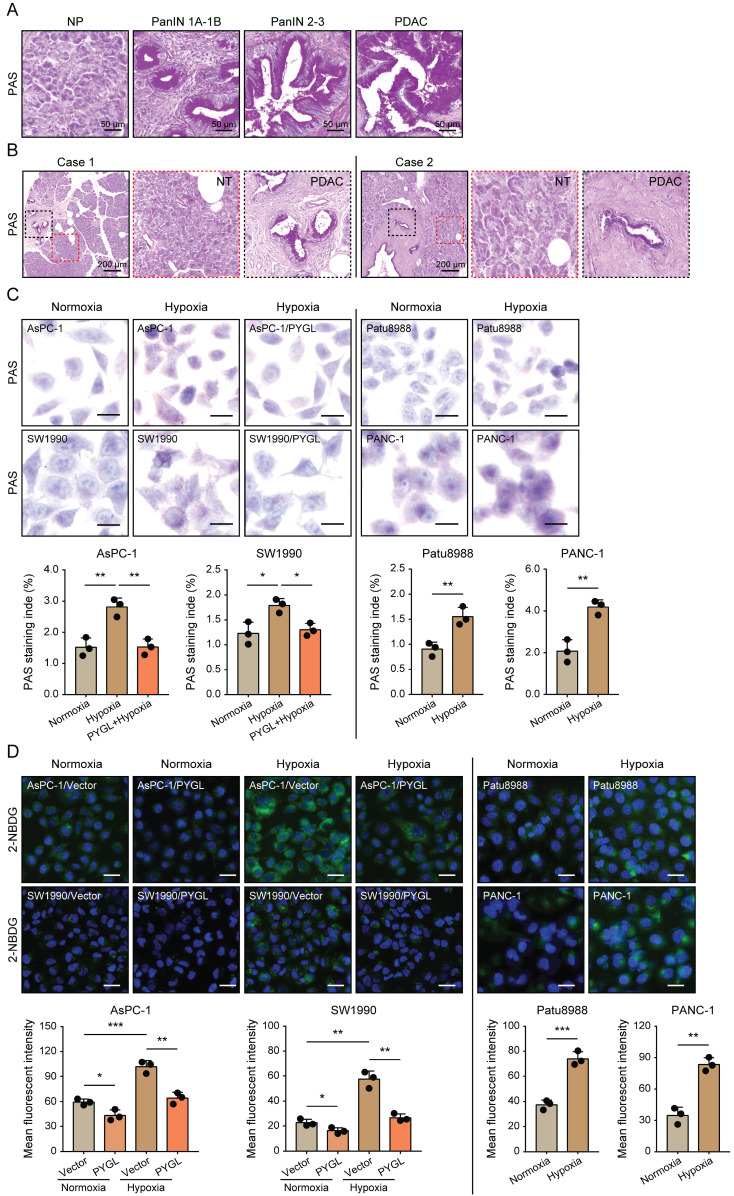
Hypoxia-induced glycogen accumulation is abrogated by PYGL. (A) PAS staining for glycogen accumulation during malignant transformation compared to normal pancreas (NP) (scale bar, 50 μm). (B) PAS staining for glycogen accumulation in the matched non-tumor (NT) tissues and PDAC tissues (scale bar, 200 μm). (C) Glycogen accumulation was detected by PAS staining in AsPC-1, SW1990, Patu8988, and PANC-1 cells as well as PYGL-overexpressing AsPC-1 and SW1990 cells exposed to hypoxia for 24 hours (scale bar, 20 μm). Quantification of PAS staining intensity (bottom panel). (D) Glycogen storage was visualized by 2-NBDG fluorescence in AsPC-1, SW1990, Patu8988, and PANC-1 cells as well as PYGL-overexpressing AsPC-1 and SW1990 cells exposed to hypoxia for 24 hours (upper panel) (scale bar, 100 μm). Quantification of 2-NBDG fluorescence (bottom panel). *P < 0.05, **P < 0.01, and ***P < 0.001.

Given that glycogen accumulation increases under hypoxic conditions 8-11, we assessed the glycogen levels in PDAC tissues characterized by hypoxic microenvironment and PDAC cells exposed to hypoxia. We observed remarkably increased glycogen content during malignant transformation from pancreatic intraepithelial neoplasia (PanINs) to PDAC compared to normal pancreas (Figure 7A). Additionally, more glycogen content was observed in PADC tissues compared with that of matched non-tumor tissues (Figure 7B). Under hypoxic conditions, glycogen accumulation was obviously induced in the detected PDAC cells as evidenced by PAS staining (Figure 7C) and 2-NBDG staining (Figure 7D). Furthermore, glycogen content was abolished by PYGL overexpression (Figure 7C-D), especially under hypoxic conditions (Figure 7D), indicating that elevated PYGL could mobilize accumulated glycogen in PDAC.

## Discussion

Cancer cells with EMT features are observed under various experimental settings and clinical situations. In view of the important role that EMT plays in PDAC metastasis and chemoresistance, we sought to identify critical regulators of PDAC EMT. In our study, we identified PYGL as a key regulator of EMT based on bioinformatics analysis and a serious of *in vitro* cellular experiments. Under hypoxic conditions, elevated PYGL mobilized accumulated glycogen to fuel glycolysis, thus inducing EMT and facilitating liver metastasis, which could be targeted by the PYGL inhibitor CP-320626 and the glycolysis inhibitor 2-DG.

Hypoxic microenvironment is especially notable in PDAC due to its prominent desmoplastic reaction 20. Glycogen accumulation has been observed under hypoxic conditions in both noncancer and cancer cell lines 8-11. In agreement with previous studies, glycogen content observed to be massively deposited in PDAC tissues and was significantly increased in PDAC cells under hypoxic conditions. The regulation of PYGL in hypoxic conditions has not been characterized, although a recent study excludes hypoxia inducible factor (HIF) as a potential player in PYGL induction in glioblastoma and breast cancer cells 8. In contrast with this report, we documented that PYGL expression was induced by hypoxia a HIF1α-dependent manner as evidenced by siRNAs targeting HIF1α and luciferase reporter assay, which may due to the different tumor context. Furthermore, we showed that, in PDAC hypoxic microenvironment, elevated PYGL could strongly mobilize glycogen accumulation to fuel glycolysis, which could be inhibited by CP-320626, in consistent with previous studies that glycogen phosphorylases act as the rate-limiting enzymes of glycogenolysis to exert their functions in several cell types, such as cancer and dendritic cells 8, 19, 21.

Emerging evidence suggests that glycolysis is closely associated with PDAC metastasis via various mechanisms, including inducing EMT 22. First, we found that PYGL promoted PDAC EMT, migration, and invasion *in vitro*, and facilitated liver metastasis *in vivo*. Next, we confirmed that the effects of PYGL in PDAC were dependent on its catalytic activity using CP-320626. At last, 2-DG was utilized to confirm the roles of PYGL-mediated enhanced glycolysis in its regulation of EMT, cell migration, and invasion. The data collectively indicate that PYGL facilitates PDAC EMT and metastasis via fueling glycolysis, establishing a link between glucose metabolism reprogramming and EMT.

The association between EMT and PDAC metastasis has been well documented, but the roles of glycolysis in PDAC EMT remain largely elusive. A recent study demonstrated that enhanced glycolysis promotes the interaction of YAP1 with TEAD1 to activate the YAP/TEAD signaling pathway and tissue-specific enhancers, thus driving neural crest EMT and cell motility 23. Several studies reported that lactate, the final product of glycolysis, makes tumor cells surrounded by an acidic microenvironment, which has been reported to induce EMT. Extracellular lactate could remodel the extracellular matrix and activate TGF-β1 to drive the expression of Snail, thereby inducing EMT and boosting cell invasion in lung cancer cells 24. Metalloproteinase (ADAM) 10/17-mediated lactate production induces EMT by upregulating the expression of mesenchymal markers in colon cancer cells 25. But our present study was not involved in the mechanism by which PYGL-mediated enhanced glycolysis induces the EMT process, which needs further study.

The studies about the roles of GPs in cancer were mainly focused on PYGB, and PYGB overexpression was observed in several cancers, including gastric cancer, ovarian cancer, non-small cell lung cancer, hepatocellular carcinoma, and colorectal cancer, and positively associated with their malignant clinical-pathological features 26-31. So far, the expression pattern of PYGL and its clinical significance in human cancers remain unclear. We for the first time showed increased PYGL protein expression in PDAC tissues, which was positively associated with tumor size, vascular invasion, and TNM stages. Simultaneously, remarkable PYGL expression in the nucleus of PDAC cells was noticed as evidenced by IHC staining. Multiple metabolism enzymes have been reported to interact with transcription factors to regulate gene expression in the nucleus 32-34. The role of nuclear PYGL in PDAC, especially in EMT, is not excluded, which is another limitation of our study.

In conclusion, our study reveals that hypoxia-induced PYGL mobilizes accumulated glycogen to fuel glycolysis, consequently facilitating PDAC EMT and metastasis, and increased PYGL predicts a poor prognosis for PDAC patients. Our findings suggest that combinedly targeting glucose metabolism at different key steps might provide a new therapeutic strategy for PDAC, a highly metastatic cancer resistant to current treatments.

## Materials and Methods

### Bioinformatics analysis

The GEO, TCGA, and Genotype-Tissue Expression (GTEx) data referenced in the study are available in a public repository from the GEO website (https://www.ncbi.nlm.nih.gov/geo/), the TCGA website (https://cancergenome.nih.gov/), and GEPIA2 website (http://gepia.cancer-pku.cn/index.html). A consensus clustering analysis of mRNA expression profiles of GEO (GSE15471, GSE16515, and GSE17891) and TCGA PDAC patients classified three subtypes: classical, quasimesenchymal (QM), and exocrine-like, based on a previously defined 62-gene signature 15. Then, both of GEO microarray gene expression profiles (46 classical and 38 QM patients) and TCGA RNA-sequencing expression profiles (31 classical and 80 QM patients) were used to identify differentially expressed mRNAs between the classical and QM subtypes. *PYGL* gene expression analysis was conducted using multiple GEO microarray gene expression data sets (GSE15471, GSE16515, GSE28735, GSE60980, GSE62165, GSE62452, GSE71729, GSE102238, GSE132956, and GSE196009) and GEPIA2 database with an overview of the RNA-sequencing data based on the TCGA and Genotype-Tissue Expression (GTEx) projects.

### Cell culture and reagents

Human PDAC cell lines AsPC-1, BxPC-3, CFPAC-1, MIA PaCa-2, PANC-1, and SW1990 were purchased from the Cell Bank of Type Culture Collection of the Chinese Academy of Sciences (Shanghai, China), Capan-1, Patu8988, and HPNE were purchased from the American Tissue Culture Collection (ATCC, Manassas, VA, USA). Hypoxia treatment was composed of 5% CO_2_, 1% O_2_, and 94% N_2_ at 37 °C. The cells were cultured in suggested standard medium containing 25 mM glucose (Hyclone, Logan, UT, USA), supplemented with 10% (v/v) fetal bovine serum (FBS, Hyclone, USA), 100 Units/mL penicillin, and 100 μg/mL streptomycin (Invitrogen, USA) as described in our previous study 35.

### RNA isolation, reverse transcription, and qRT-PCR

Total RNA was isolated from cultured cells using Trizol reagent (Takara Bio, Japan) following the manufacturer's instructions and was subsequently reverse-transcribed into cDNA with a prime Script RT reagent kit (Takara Bio, Japan). For gene expression analysis, qRT-PCR was performed with primers using 2 × SYBR Green qPCR Master Mix (Bimake, Shanghai, China) on an ABI7500 instrument (Applied Biosystems). The primer sequences were listed as follows: *18sRNA* (forward: 5'-TGCGAGTACTCAACACCAACA-3', reverse: 5'-GCATATCTTCGGCCCACA-3'), *PYGL* (forward: 5'-AAATGCCTGTGATGAGGCCA-3', reverse: 5'-CATGAATTCTGGGCGGGACT-3'),* CDH1* (forward: 5'-TCAGGGTCGGTTGGAAATC-3', reverse: 5'-AATGCCGCCATCGCTTAC-3'), *CDH2* (forward: 5'-GTCAGCAGAAGTTGAAGAAATAGTG-3', reverse: 5'-GCAAGTTGATTGGAGGGATG-3'), *Vimentin* (forward: 5'-TCTTCCAAACTTTTCCTCCC, reverse: 5'-AGTTTCGTTGATAACCTGTCC-3'), *Occludin* (forward: 5'-ACCAATGCTCTCTCAGCCAG-3', reverse: 5'-AGGCAAAGATGGCAATGCAC-3'). 18sRNA was amplified as an internal control.

### Western blotting analysis

Western blotting analysis was performed according to the established protocols as previously described 35. The primary antibodies used in this study were as follows: PYGL (1:1,000, 15851-1-AP), GYS1 (1:2,000, 10566-1-AP), GBE1 (1:3,000, 20313-1-AP), PYGM (1:5,000, 19716-1-AP), PYGB (1:1,000, 12075-1-AP), E-cadherin (1:1,000, 20874-1-AP), Occludin (1:2,000, 13409-1-AP), N-cadherin (1:2,000, 22018-1-AP), and Vimentin (1:2,000, 10366-1-AP) from Proteintech, and β-actin (1:5,000, ab008) from MultiSciences.

### Lentiviral transduction

For overexpression, lentiviruses overexpressing PYGL (GeneCopoeia, Guanghzou, China) were transduced into the target PDAC cells according to the manufacturer's instructions. The transfected cells were selected with 10 μg/mL blasticidin for two weeks. For knockdown, short hairpin RNA (shRNA) plasmids targeting PYGL were purchased from Shanghai Genechem Co. and shRNA sequences were listed as follows: shPYGL-1: 5'-TACCAGCTTGGATTGGATATA-3'; shPYGL-2: 5'-CCAGGATATCACATGGCCAAA-3'; shPYGL-3: 5'-GCCTATGTCAAGTGTCAAGAT-3'. Lentiviruses were packaged and produced using a three-plasmid expression system (pCDH-PACK-GAG, pCDH-PACK-REV, and VSV-G), and transfected into target cell lines as described previously 36. The transfected cells were then selected with 5 µg/mL puromycin for 2 weeks. The efficiency of PYGL overexpression or knockdown was confirmed by qRT-PCR and western blotting assays.

### siRNA transfection

The siRNAs targeting HIF1α and a negative control siRNA were purchased from GenePharm (Shanghai, China) and were used to transfect AsPC-1 and PANC-1 cells as previously described 37. 48 hours later, the cells were harvested for validation of the siRNA transfection efficiency and further experiments. siRNA sequences were listed as follows: siHIF1α-1 (HIF1α-Homo-1168: 5'-GAUGAAAGAAUUACCGAAUTT-3'), siHIF1α-2 (HIF1α-Homo-2090: 5'-CUCCCUAUAUCCCAAUGGATT-3').

### Immunofluorescence (IF) staining

The indicated cells were seeded on the confocal dishes overnight, washed with 1 × PBS three times, fixed in 4% paraformaldehyde for 30 min, blocked with 1% bovine serum albumin for 1 h and incubated with antibodies against E-Cadherin (1:150, Proteintech, 20874-1-AP), N-Cadherin (1:150, Proteintech, 22018-1-AP), and Vimentin (1:150, Proteintech, 10366-1-AP) antibodies for 1 h at room temperature (RT). Then, the cells were incubated with Alexa Fluor 594-conjugated secondary antibodies (Molecular Probes, USA) for 1 h at RT and subsequently mounted with the DAPI-containing mounting medium (Beyotime, Shanghai, China). The images were taken with a laser scanning confocal microscope (Leica Microsystems AG).

### CCK-8 and colony formation assays for cell proliferation

For CCK-8 assay, 1-2 × 10^3^ cells were planted in 96-well plates in triplicate and observed for 5 days. The culture medium was removed and 100 μL of CCK-8 at a dilution of 1:10 (v/v) in the medium was added to each well at the indicated time points. After incubated for 60 min in the cell incubator, the plates were measured at 450 nm using a multifunctional microplate reader (Bio-Rad Laboratories, Hercules, CA). For colony formation assay, 1 × 10^3^ cells were planted in 6-cm plates and incubated for 10-14 days. Then, cells were washed with 1 × PBS twice, fixed with 4% paraformaldehyde for 30 min, and stained with 0.2% crystal violet for 1 h. Colonies larger than 100 μm in diameter for each plate were counted.

### SA-β-gal staining

A Cellular Senescence Detection Kit (Cell Signaling Technology) was used to detect senescent cells according to the manufacturer's instructions. Images were obtained with an inverted light microscope and 5 independent fields were quantified using ImageJ software.

### Transwell migration and invasion assays

The migration and invasion assays were performed using 8-μm-pore Transwell chamber (JET BOIFIL, Guangzhou, China). For migration assay, 5 × 10^4^ cells in 200 μL serum-free medium were seeded into the upper chambers and migrated for 24 h. For invasion assay, 1 × 10^5^ cells in 200 μL serum-free medium were seeded into the chambers coated with Matrigel (BD Biosciences, Franklin Lakes, NJ, USA) and invaded for 48 h. The bottom chambers contained 800 μL of the corresponding medium with 10% FBS. Then the cells were fixed and stained with crystal violet (Beyotime, Shanghai, China) for 30 min, followed by removing non-invading cells on the top surface of the chambers with cotton swabs. Finally, stained cells were counted in five randomly selected fields under an inverted light microscope.

### *In vivo* metastatic assay

2 × 10^6^ cells (PYGL-overexpressing AsPC-1 cells, PYGL knockdown Patu8988 cells, and their vector control cells) were resuspended in 25 μL 1 × PBS and injected into the spleen of Balb/c nude mice for the establishment of the liver metastatic model. Mice were sacrificed 4 weeks post-injection and the livers were dissected. To examine the liver metastases foci, liver tissues were fixed with 4% paraformaldehyde, embedded in paraffin, and stained with hematoxylin and eosin (H&E). Liver metastasis was evaluated by counting the metastatic foci in 5 randomly selected fields. The mice were manipulated and housed according to protocols approved by the Jiangsu University Animal Care Commission. All the mice received humane care according to the criteria outlined in the Guide for the Care and Use of Laboratory Animals prepared by the National Academy of Sciences and published by the National Institutes of Health.

### Glycogen staining

In the current study, we used two different approaches to label glycogen. (i) Glycogen was detected in cells and tumor tissues with a standardized PAS staining technique. For cell lines, cells were fixed with 4% paraformaldehyde for 15 min. For tumor tissues, slides were deparaffinized and rehydrated to deionized water. Samples were incubated in periodic acid (Sigma-Aldrich) for 5-10 min at RT, rinsed in water, and bathed with Schiff's reagent for 15 min. After washed under running water for 5 min, the samples were counterstained with hematoxylin, and for tumor tissues, slides were mounted in xylene-based mounting media. Images were captured using a Zeiss Axiovert 135 microscope. (ii) 2-NBDG, a fluorescent derivative of glucose acting as a substitute of glucose competitively integrated to form glycogen, was used for glycogen labelling in live PDAC cells. Briefly, cells were seeded on the confocal dishes in 24-well plates. The next day media was removed from the cells and replaced with glucose-free DMEM or RPMI 1640 (Life Technologies). After 3 hours, the cells were incubated in no-glucose media supplemented with 100 μM 2-NBDG (Cayman, 11046) for 45 min and then imaged using a laser scanning confocal microscope (Leica Microsystems AG).

### Glycogen quantification

Glycogen levels were measured using a Glycogen Assay Kit (BioVision) according to the manufacturer's instructions. Briefly, the cells were homogenized with 200 μL of dH_2_O on ice and then boiled for 5 min. Homogenates were spun at 13,000 rpm for 5 min and supernatants were assayed for glycogen content. Results were normalized by protein content or cell number.

### ECAR and lactate production assay

ECAR was measured using the Seahorse XF96 Flux Analyzer (Seahorse Bioscience, Billerica, Massachusetts, USA) and lactate production was measured using a Lactate Assay Kit (BioVision) following the manufacturer's instructions as detailly described in our previous study 35.

### Luciferase reporter assay

PYGL promoter sequence was obtained from USCS genomic browser. The 1000-base promoter region of PYGL was cloned into pGL4-basic reporter vector. The promoter sequence was uploaded to the JASPAR database to predict HIF1A/ARNT binding sites and the threshold was set to 80%. The mutant PYGL promoter was constructed with HIF1A/ARNT binding sites mutated at PYGL promoter region. The PYGL promoter reporters were co-transfected with control plasmid pRL-TK into AsPC-1 and PANC-1 cells. The promoter activity was determined by a Dual-Luciferase Assay (Promega, Madison, WI).

### IHC staining

A paraffin-embedded tissue microarray (OD-CT-DgPan01-006), containing 90 pairs of pancreatic cancer tissues and adjacent non-tumor tissues, was purchased from Shanghai Outdo Biotech Inc (Shanghai, China). IHC staining was performed using a two-step protocol and scoring was conducted based on both the ratio and intensity of the staining as previously described in detail 38. Primary antibody used for IHC staining was PYGL (1:200, Proteintech, 15851-1-AP).

### Statistical analysis

Data analysis was performed using GraphPad Prism 7.0 software and SPSS 19.0 software. Quantitative variables were analyzed by the two-tailed Student t-test or analysis of variance. Pearson's correlation analysis was used to determine the correlation between two indicated molecular expression. Kaplan-Meier survival curves were created using the log-rank test to compare the high PYGL group with the low PYGL group. The results were considered statistically significant when *P < 0.05, **P < 0.01, and ***P < 0.001.

## Supplementary Material

Supplementary figures and tables.

## Abbreviations

PDAC: pancreatic ductal adenocarcinoma
EMT: epithelial-mesenchymal transition
PYGL: glycogen phosphorylase L
TCGA: the Cancer Genome Atlas
GEO: Gene Expression Omnibus
GSEA: gene set enrichment analysis
qRT-PCR: quantitative real-time PCR
IF: immunofluorescence
2-NBDG: 2-(N-(7-nitrobenz-2-oxa-1,3-diazol-4-yl) amino)-2-deoxyglucose
ECAR: extracellular acidification rate
IHC: immunohistochemistry
2-DG: 2-deoxy-D-glucose
PAS: periodic acid Schiff
HIF1α: hypoxia inducible factor 1α
SA-β-gal: senescence-associated β-galactosidase
