# Supplementary material for: PYGL-mediated glucose metabolism reprogramming promotes EMT phenotype and metastasis of pancreatic cancer
Source: Int J Biol Sci. 2023 Mar 21;19(6):1894–909. doi: 10.7150/ijbs.76756 (PMC10092766; doi:10.7150/ijbs.76756)
Supplement: Supplementary file 1 — Supplementary figures and tables. [file ijbsv19p1894s1.pdf]

Supplementary Material

Supplementary figures

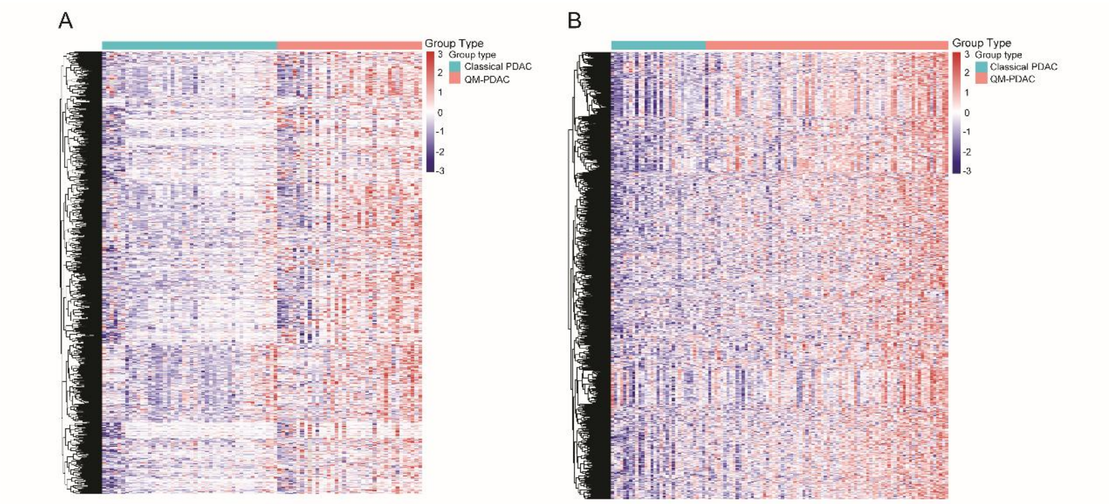

Figure S1. Pancreatic cancer patients with molecular profiling data were classified into epithelial and mesenchymal subtypes for further analysis. Heatmap of significantly upregulated genes in mesenchymal subtypes compared with epithelial subtypes in the GEO (A) and TCGA (B) datasets.

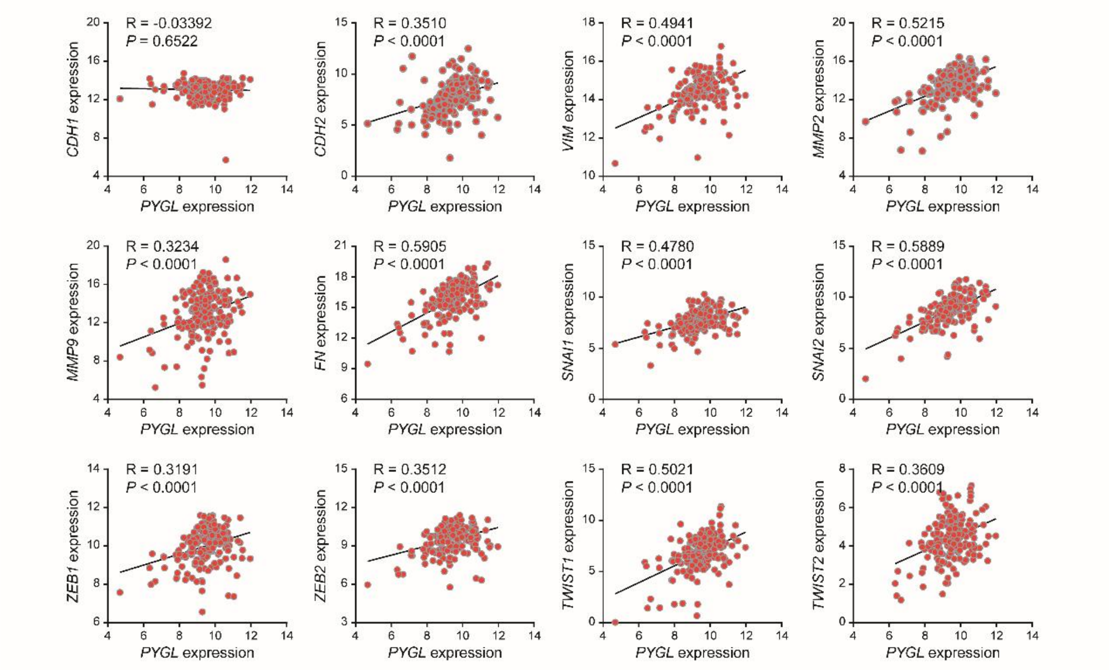

Figure S2. The correlation between *PYGL* expression and EMT markers at mRNA level revealed by analyzing the TCGA database.

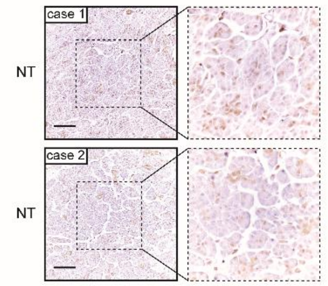

Figure S3. The expression pattern of PYGL protein in the non-tumor (NT) tissues with relatively higher PYGL expression in tissue microarray revealed by IHC staining and representative IHC images of PYGL in NT tissues (scale bar, 50 μm).

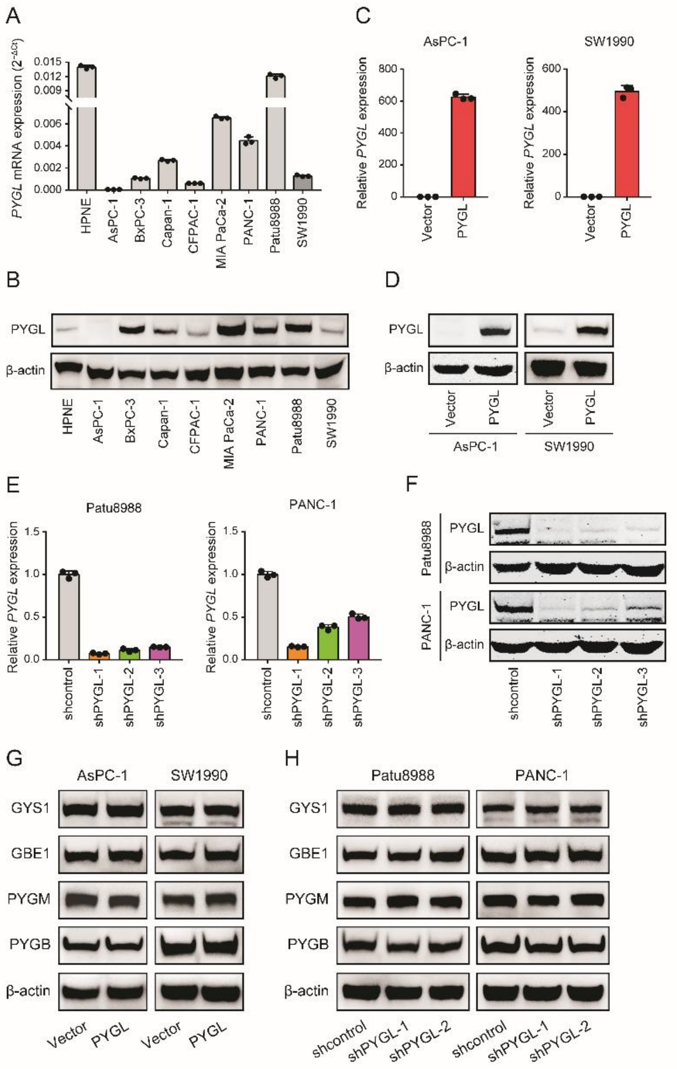

Figure S4. PYGL expression in PDAC cell lines and validation of PYGL overexpression and knockdown efficiency. (A-B) PYGL expression in immortalized normal pancreatic ductal cell

(HPNE) and PDAC cell lines examined by qRT-PCR (A) and western blotting (B). (C-D) PYGL overexpression efficiency in AsPC-1 and SW1990 cells validated by qRT-PCR (C) and western blotting (D). (E-F) PYGL knockdown efficiency in Patu8988 and PANC1 cells validated by qRT-PCR (E) and western blotting (F). (G-H) The expression of other key enzymes of the glycogen metabolism, including GYS1, GBE1, PYGB, and PYGM, following PYGL overexpression (G) or knockdown (H) examined by western blotting.

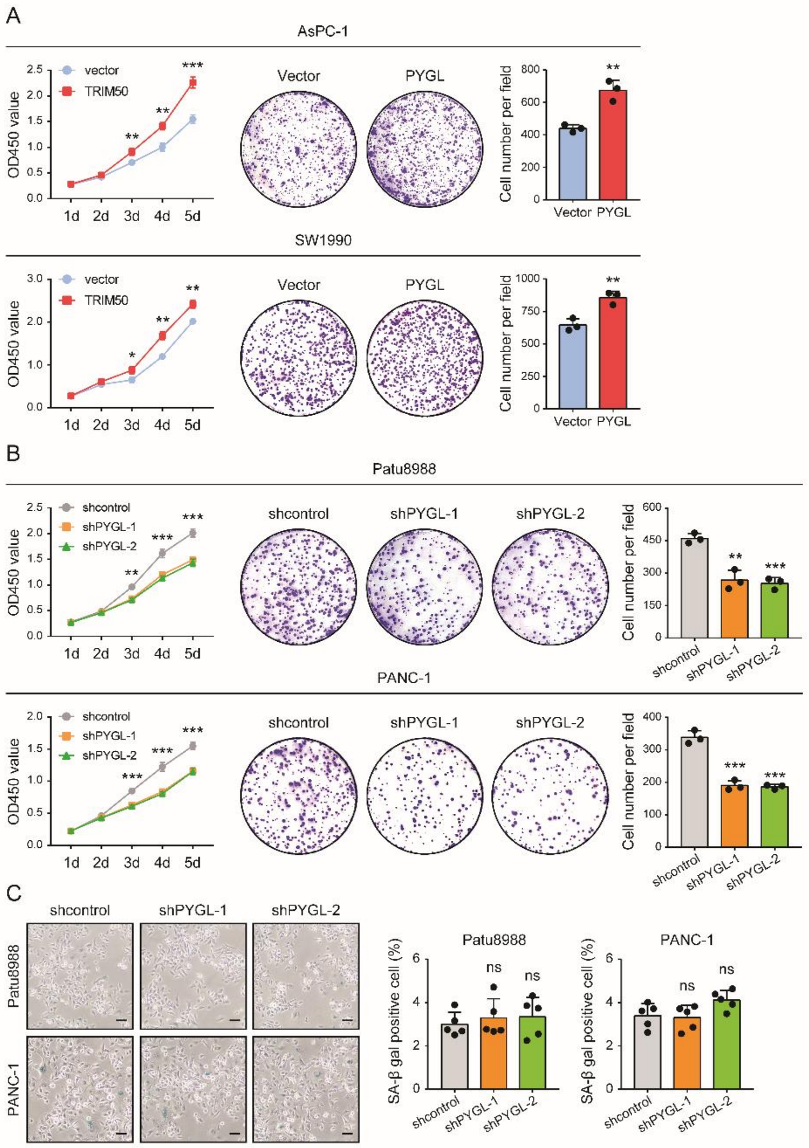

Figure S5. PYGL promotes PDAC cell proliferation. (A) The effects of PYGL overexpression on cell proliferation in AsPC-1 and SW1990 cells revealed by CCK-8 (left panel) and colony formation assays (right panel). (B) The effects of PYGL knockdown on cell proliferation in Patu8988 and PANC-1 cells revealed by CCK-8 (left panel) and colony formation assays (right

panel). (C) The effects of PYGL knockdown on cellular senescence in Patu8988 and PANC-1 cells revealed by SA-β-gal staining (scale bar, 200 μm). ns, no significance, *P < 0.05, **P < 0.01, and ***P < 0.001.

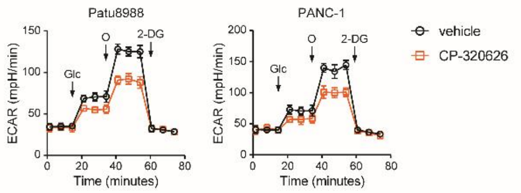

Figure S6. The effects of CP-320626 on glycolysis of Patu8988 and PANC-1 cells were reflected by ECAR using the Seahorse SF96 Extracellular Flux Analyzer (Glc, glucose; O, oligomycin).

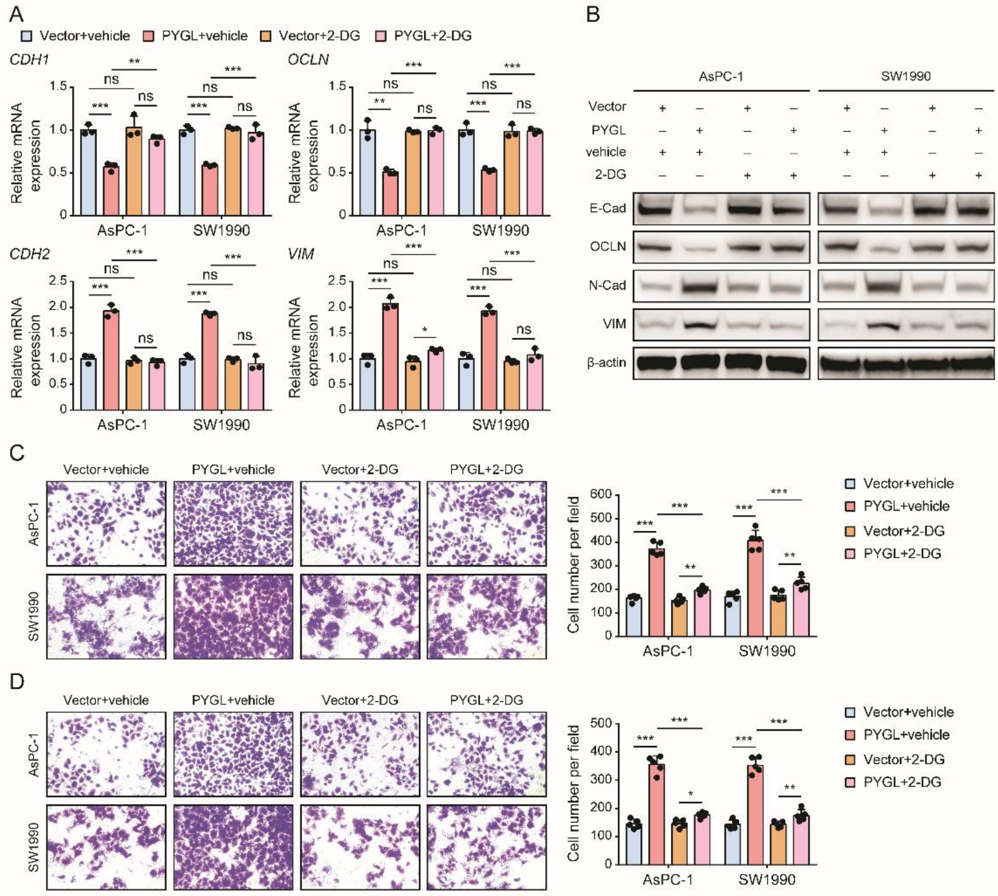

Figure S7. Enhanced glycolysis is responsible for the promoting effects of PYGL on EMT, cell migration, and invasion. (A) The mRNA expression of EMT markers (*CDH1*, *OCLN*, *CDH2*, and *VIM*) in PYGL-overexpressing and vector control cells treated with or without 2-DG (2 mM for 24 hours) examined by qRT-PCR. (B) The protein expression of EMT markers (E-Cad,

OCLN, N-Cad, and VIM) in PYGL-overexpressing and vector control cells treated with or without 2-DG (2 mM for 24 hours) examined by western blotting. (C-D) Transwell migration (C) and invasion (D) assays of PYGL-overexpressing and vector control cells treated with or without 2 mM 2-DG (magnification, 200 ×). ns, no significance, **P* < 0.05, ***P* < 0.01, and ****P* < 0.001.

Supplementary tables

Table S1. EMT-related genes with significant prognostic value

**Gene Symbol**	**Description**	***P* value**
KIF23	Kinesin Family Member 23	0.000291246
PYGL	Glycogen Phosphorylase L	0.000514503
DIAPH3	Diaphanous Related Formin 3	0.000777197
FOSL1	FOS Like 1, AP-1 Transcription Factor Subunit	0.003599949
C16orf74	Chromosome 16 Open Reading Frame 74	0.019444231
HIST1H2BJ	H2B Clustered Histone 11	0.02017281
LOX	Lysyl Oxidase	0.022957134
ARSI	Arylsulfatase Family Member I	0.023758968
CABYR	Calcium Binding Tyrosine Phosphorylation Regulated	0.030640603
SULF2	Sulfatase 2	0.030700529
CEBPB	CCAAT Enhancer Binding Protein Beta	0.034549891
TWIST1	Twist Family BHLH Transcription Factor 1	0.034593791
KRT14	Keratin 14	0.037196449
SNCG	Synuclein Gamma	0.048006318
SNAI2	Snail Family Transcriptional Repressor 2	0.048187917
C17orf53	Chromosome 17 Open Reading Frame 53	0.048864299

Table S2. Correlations between PYGL expression and clinicopathologic parameters in PDAC patients

Clinicopathological parameter	Total 90	Expression of PYGL		*P* value
		Low (n = 35)	High (n = 55)	
**Age (years)**				
< 60	41	18	23 (63.6)	0.394
≥ 60	49	17	32 (76.3)	
**Gender**				
Male	57	21	36	0.657
Female	33	14	19	
**Tumor size**				
≤ 5 cm	55	27	28	0.008*
> 5 cm	34	7	27	
**Tumor grade**				
I	1	1	0	0.227
II	56	24	32	
III	22	5	17	
IV	1	1	0	
**Lymphatic metastasis**				
Absent	48	23	25	0.167
Present	33	10	23	
**Vascular invasion**				
Absent	52	25	27	0.049*
Present	38	10	28	
**Distant metastasis**				
Absent	88	35	53	0.519
Present	2	05	2	
**TNM stage**				
I	40	23	17	0.005*
II	47	11	36	
IV	2	0	2	

[a]The bold number represents the *p*-values with significant differences.

[b]*P* value was calculated by χ^2 test or Fisher's exact test.
